# Non‐gonadal expression of piRNAs is widespread across Arthropoda

**DOI:** 10.1002/1873-3468.15023

**Published:** 2024-10-02

**Authors:** Takahisa Yamashita, Krystian Komenda, Rafał Miłodrowski, Dominik Robak, Szymon Szrajer, Tomasz Gaczorek, Guillem Ylla

**Affiliations:** ^1^ Laboratory of Bioinformatics and Genome Biology, Faculty of Biochemistry, Biophysics and Biotechnology Jagiellonian University Krakow Poland; ^2^ Doctoral School of Exact and Natural Sciences Jagiellonian University Krakow Poland

**Keywords:** Argonaute, Arthropods, bioinformatics, piRNAs, PIWI, small RNAs

## Abstract

PIWI‐interacting RNAs (piRNAs) were discovered in the early 2000s and became known for their role in protecting the germline genome against mobile genetic elements. Successively, piRNAs were also detected in the somatic cells of gonads in multiple animal species. In recent years, piRNAs have been reported in non‐gonadal tissues in various arthropods, contrary to the initial assumptions of piRNAs being exclusive to gonads. Here, we performed an extensive literature review, which revealed that reports on non‐gonadal somatic piRNA expression are not limited to a few specific species. Instead, when multiple studies are considered collectively, it appears to be a widespread phenomenon across arthropods. Furthermore, we systematically analyzed 168 publicly available small RNA‐seq datasets from diverse tissues in 17 species, which further supported the bibliographic reports that piRNAs are expressed across tissues and species in Arthropoda.

## Graphical abstract

PIWI‐interacting RNAs (piRNAs) are known to protect the germline genome from transposable elements. Recently, evidence for their non‐gonadal expression has begun to emerge in some species. In this Perspective article, we combine an extensive literature review with a large analysis of transcriptomic data across arthropod species to investigate non‐gonadal piRNA expression. We show that non‐gonadal piRNA expression is a widespread phenomenon in Arthropoda.

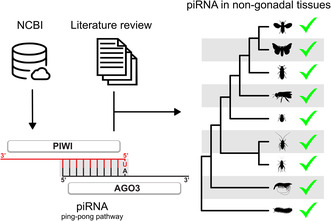

## Abbreviations

**AGO1, 2, 3**, Argonaute1, 2, 3

**AUB**, Aubergine

**Cbl**, E3 ubiquitin‐protein ligase CBL

**c‐Fos**, Fos Proto‐Oncogene, AP‐1 Transcription Factor Subunit

**Fem**, feminizer

**Gtsf1**, gametocyte specific factor 1

**KRIMP**, Krimper

**lnc‐RNA**, long non‐coding RNA

**Masc**, masculinizer

**miRNA**, micro RNA

**NBR**, Nibbler

**ncRNA**, non‐coding RNA

**piRNA**, PIWI‐interacting RNAs

**PIWI**, P‐element induced wimpy testis

**siRNA**, short interfering RNAs

**TEs**, transposable elements

**VAS**, Vasa

**Yb**, female sterile (1) Yb

**ZUC**, Zucchini

## A brief history of piRNAs

In 1993, a type of small noncoding RNAs was discovered with a powerful potential to regulate mRNAs expression at the post‐transcriptional level [1, 2]. With a length of 21–23 nucleotides (nts), these small RNAs, named micro RNAs (miRNAs), were subsequently found to be important regulators of the gene expression regulatory network across animals for their role in mRNAs repression [1]. A few years later, in 1998, another type of small noncoding RNAs was identified with genetic interference capacity, the short interfering RNAs (siRNAs) [3]. These findings put the spotlight on small noncoding RNAs as small molecules with powerful roles in gene expression regulation.

In 2001, Aravin *et al*. [4], reported a new type of small RNAs capable of silencing transposable elements (TEs) in the germline of *Drosophila melanogaster*. These were P‐element induced wimpy testis (PIWI)‐interacting RNAs, abbreviated as piRNAs, although this name would not be coined until 5 years later when a type of small RNAs was identified to bind to the protein PIWI and to be abundant in the *D. melanogaster* germline and testes of mammals [5, 6, 7, 8, 9]. In these initial articles, and the ones that followed, piRNAs were found to play an important role in protecting the germ cells' genome against the activity of mobile genetic elements such as transposable elements [10, 11]. Despite the initial reports of exclusive piRNA expression in germ cells, piRNA expression was later found to also occur in somatic cells of the gonads of some animals such as in *D. melanogaster*'s somatic ovarian follicle cells [12, 13, 14].

More recently, a growing number of studies in a wider range of experimental organisms have reported instances of piRNA expression outside the gonads of flies and mammals. For example, a study in mollusks found that piRNAs and piRNA‐related proteins are ubiquitously expressed in the oyster *Crassostrea gigas* and in the great pond snail *Lymnaea stagnalis* [15]. In pan‐arthropods, somatic piRNA expression was shown to be present in different lineages including insects [16], which was in contrast to the previously prevailing assumption, based on initial findings from the genetic model *D. melanogaster*, that insect piRNA expression was restricted to the gonads.

Here, we review the literature to provide a comprehensive overview of the current knowledge on non‐gonadal piRNA expression in arthropods. Through this review, we identified numerous instances of piRNAs being documented in non‐gonadal tissues across species from diverse orders. Furthermore, based on analysis of 168 publicly available small RNA‐seq datasets of 17 arthropod species, we provide new evidence that piRNAs are expressed in different non‐gonadal tissues across almost all arthropod orders analyzed. Thus, it appears that non‐gonadal piRNA expression is a common occurrence in Arthropods, rather than an exception.

## piRNA biogenesis, insights from *Drosophila melanogaster*

Arguably, the piRNA biogenesis pathways have been best characterized in the fruit fly *D. melanogaster*. In this fly, as in most of the other animal models studied, piRNA biogenesis is divided into two pathways, the primary pathway and the secondary pathway, also known as the ping‐pong pathway. In general, both pathways are active in germ cells, while the primary pathway has also been shown to be active in somatic cells of the gonads [17, 18, 19]. In this species, three different proteins bind to piRNAs, AGO3, PIWI, and AUB, all belonging to the Argonaute superfamily, a superfamily that also includes AGO1 and AGO2 that mediate in the miRNA and siRNA pathways, respectively. PIWI is considered to participate in the piRNA biogenesis only through the primary pathway, while AUB and AGO3 only in the ping‐pong cycle.

**Fig. 1:**
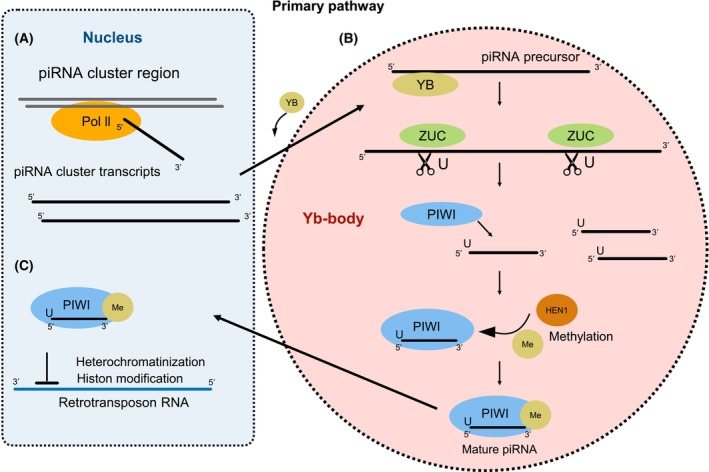
Overview of the primary piRNA biogenesis pathway based on *Drosophila melanogaster*. (A) The piRNA cluster transcripts are transcribed by Polymerase II, and exported to the cytoplasmic Yb‐body by YB. (B) In the Yb‐body, the piRNA precursor transcripts are cleaved by ZUC. Then, the cleaved fragments are loaded onto PIWI and methylated by HEN1 resulting in mature piRNAs. (C) Mature piRNAs exported to the nucleus can bind to retrotransposon RNA transcripts causing heterochromatinization or histone modification resulting in expression repression.

The primary pathway starts with the transcription of long transcripts by Polymerase II (Fig. 1A). Subsequently, YB binds to the transcripts and transfers them to the Yb bodies, membraneless organelles in the cytoplasm surrounded by mitochondria that contain piRNA biogenesis proteins [16, 20, 21]. The translocated transcripts are cleaved into ~ 28 nts fragments by Zucchini (ZUC) [22, 23] (Fig. 1B). The released ~ 28 RNA fragments, with predominant 5′U [24], are incorporated into the P‐element induced wimpy testis (PIWI) and subsequently methylated at the 3′ end by HEN1, producing the mature primary piRNA [23]. Mature piRNAs can recognize complementary RNAs, often transposons, and induce trimethylation of histone H3 and heterochromatinization by modification of histone H1 repressing the transposons at the transcriptional level [14, 25] (Fig. 1C).

**Fig. 2:**
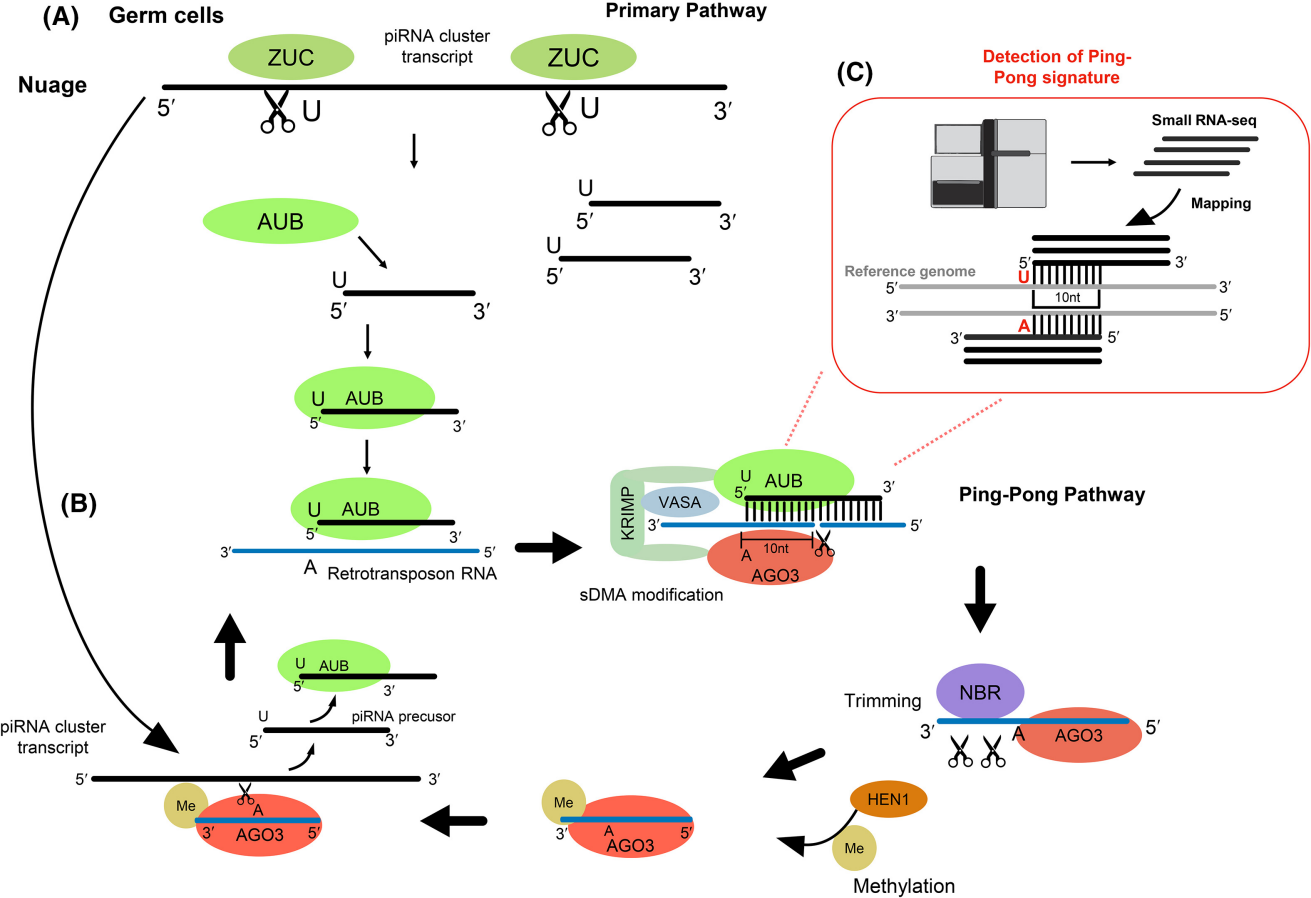
Overview of the secondary piRNA biogenesis pathway (also known as ping‐pong cycle) based on *Drosophila melanogaster*. (A) In the nuage region of germ cells, piRNA transcripts cleaved by ZUC might be loaded onto AUB. (B) AUB uses piRNA to identify retrotransposon RNAs and cleave them. The resulting RNA fragments are loaded onto AGO3 recruited by KRIMP and together with VASA. The 3′ end of the loaded RNA is trimmed by NBR and methylated by HEN1, resulting in a mature secondary piRNA. The secondary mature piRNA is complementary to the primary piRNA cluster transcript, which can target and trigger the production of piRNAs like the original primary one, closing this way the ping‐pong loop. (C) The presence of ping‐pong piRNAs can be robustly determined by the detection of the ping‐pong signature, which consists of small RNA‐seq reads of the length of the piRNAs, aligned in complementary strands of the genome with 10nts 5′‐to‐5′ overlap. These pairs of reads derive from the primary and secondary piRNAs produced during the ping‐pong cycle.

The secondary pathway (Fig. 2), also known as the ping‐pong pathway, was initially reported to be restricted to the germline in *D. melanogaster* [14]. This ping‐pong pathway occurs in the nuage [26], a membraneless organelle located near the germline nuclear membrane [27]. The piRNA fragments produced through the primary pathway are incorporated into Aubergine (AUB), which belongs to the PIWI clade and uses the piRNA sequence to identify and bind the target RNA molecules [28] (Fig. 2A). Next, Krimper (KRIMP) and Vasa (VAS) participate in the recruitment of AGO3 [29, 30], and the target RNA is cleaved at the position corresponding to the complementary of the 10th nucleotide of the primary piRNA. The 3′ end of the target sequence is then trimmed by Nibbler (NBR) and methylated by HEN1, generating a mature secondary piRNA bound to AGO3 that is complementary to the initial precursor piRNA transcript. AGO3 uses the secondary piRNA to identify the initial precursor piRNA [31] and triggers the production of more piRNAs like the initial primary piRNA, which closes the ping‐pong loop [23] (Fig. 2B). As a result, this cycle increases the production of those piRNAs that have RNA targets in the cell [32, 33]. It is worth noting that AUB, a paralogue of PIWI that is exclusive to flies [34], is required for *D. melanogaster*'s ping‐pong cycle [18, 28, 35]. Other insects do not possess the *aub* gene, but they often have more than one *piwi* paralogue which might perform an equivalent role to *aub* in flies in the ping‐pong pathway.

The activity of AGO3 cleaving the target RNA at the position that corresponds to the 10 nt of the primary piRNA to which is bound, leads to 10 nucleotides 5′‐to‐5′ complementarity with the primary and secondary piRNAs. Furthermore, because the primary piRNAs are enriched by U at the 5′ end and there is the 10 nts complementarity between primary and secondary piRNAs, the secondary ones display an enrichment of A at the 10th position. This characteristic 10 nt complementarity between primary and secondary piRNAs with the primary displaying 5′U and secondary 5′10A has been named “ping‐pong signature” [28] and it is considered a hallmark of ping‐pong piRNA expression. Based on that, the identification of a significant number of small RNA‐seq reads mapped into the genome that display 10 nucleotides 5′‐to‐5′ overlap can be leveraged to determine the presence of ping‐pong piRNA expression from small RNA‐seq data [36] (Fig. 2C).

## Canonical roles of piRNAs in arthropods

The roles of piRNAs have largely been associated with the protection of the genome against mobile genetic elements in germ cells, such as against TEs, which can form large fractions of animal genomes [37]. While the activity of transposons might have contributed to the evolution of organisms through the acquisition of genetic diversity and dynamic changes in genomes [38], they often disrupt gene expression and cause gene mutations. This is critical in germ cells, as their genome can be transmitted to the progeny, and hence, piRNA action is particularly important in the germline [14, 28, 36].

Genomic regions containing transposons are often transcriptionally repressed by DNA methylation and repressive histone modifications. Additionally, piRNAs also participate in the protection of the genome against transposable elements, by repressing transposon activity at both transcriptional and post‐transcriptional levels through heterochromatinization and RNA silencing, respectively [39, 40].

## Non‐gonadal expression of piRNAs in insects

Since the 19^th^ century, *D. melanogaster* has been among the most widely used models for genetic research and, as such, is often the preferential representative for studying insect biology. Other insects also gained importance as research models, in part, due to their economic impact such as the silkworm *Bombyx mori*, the red flour beetle *Tribolium castaneum*, or the honeybee *Apis mellifera*. All of these commonly studied insect species belong to the superorder Holometabola, distinguished by their complete metamorphosis [41]. Given the substantial volume of research dedicated to these species, we provide a specific section dedicated to each of the four largest orders of holometabolous insects: Diptera, Lepidoptera, Hymenoptera, and Coleoptera.

The holometabolous metamorphosis emerged from the ancestral hemimetabolous metamorphosis, characterized by gradual changes along post‐embryonic development [42]. Several orders of insects still maintain this ancestral mode of development. While there is less available genetic data for insect species with the hemimetabolous developmental mode, the pea aphid *Acyrthosiphon pisum*, the cockroach *Blattella germanica*, and the cricket *Gryllus bimaculatus* stand out as the best‐studied hemimetabolous species at a genetic level [42, 43, 44, 45]. Therefore, we include specific sections dedicated to the orders encompassing these species: Hemiptera, Blattodea, and Orthoptera.

Outside Insecta, genetic data on other arthropod species are very scarce. Due to the limited reports on these non‐insect arthropod lineages, including Crustacea, Myriapoda, and Chelicerata, they are discussed together in the final part of this literature review section.

### Diptera

In *D. melanogaster*, expression of piRNAs has been reported in the germ cells and somatic cells of the ovaries [19] and testes [26]. In the ovaries, piRNAs are expressed in both germ cells and somatic cells of the follicular epithelium. The genes *piwi*, *aub*, and *AGO3* are all expressed in germ cells, while only *piwi* is expressed in somatic cells of the gonads, in which only the primary piRNA pathway is reportedly active [18, 19].

The piRNAs in the *D. melanogaster* ovaries have been widely reported to play the canonical role of suppressing retrotransposons [14]. The knockdown experiments of *aub* and *AGO3* in germ cells cause female infertility and abnormalities in embryonic axis determination [46]. *AGO3* mutants in male testes showed a reduction in germline stem cells, suggesting a role of piRNAs in the regulation of stem cells [47]. Interestingly, ovarian piRNAs also have the potential to regulate mRNAs [48, 49, 50, 51], such as *c‐Fos*, a factor involved in cell proliferation and differentiation [49], and *Cbl*, involved in hematopoietic stem cell maintenance [50]. Recently, male testes piRNAs have also been shown to silence endogenous genes during spermatogenesis [52].

Despite the still common view that *D. melanogaster* piRNA expression is restricted to the gonads, there is growing evidence that non‐gonadal somatic tissues also produce piRNAs. For example, piRNA expression has been detected in heads, imaginal discs [53], embryos [54, 55], and fat bodies, where *piwi* mutants display decreased piRNAs expression, increased TE expression, and a shorter lifespan [56]. It has also been shown that the *D. melanogaster* brain expresses *aub* and *AGO3*, and the loss of expression of these genes was associated with an increase in transposon activity, implying a potential activity of piRNAs in the brain [57].

The expression of piRNAs has recently been studied in the ovaries of other fly species. One study on 119 *Drosophila* species concluded that ovarian somatic expression of primary piRNAs in the *Drosophila* genus is widely conserved and essential for the ovaries [58]. Another recent article reported that *Drosophila* species from the *obscura* group may have lost *Yb*, and that *Drosophila eugracilis* may have independently lost *Yb* in addition to *AGO3* [59]. Despite the absence of these genes, both species produce piRNAs antisense to TEs in ovarian somatic cells.

Non‐gonadal piRNA expression has also been detected in other fly species. For example, in *Musca domestica* and *Drosophila virilis*, ping‐pong piRNAs were detected in the female thorax, which contrasted with a lack of detection of ping‐pong piRNAs in *D. melanogaster* thorax in the same study [16].

In mosquitoes, piRNAs are expressed in the ovaries and testes. Additionally, high expression has also been reported in somatic cells of adult males and larvae [60], and in the thorax of female specimens [16]. Most of the functions of piRNAs described in mosquitoes are related to the regulation of transposon and viral RNAs, but some piRNAs also appear to be involved in the degradation of lnc‐RNA [61] and maternal mRNAs [62].

In the mosquito *Aedes albopictus*, piRNA expression has been identified in somatic cells in response to viral infection [63]. Indeed, there are numerous reports of the antiviral role of piRNAs in multiple mosquito species including *Aedes aegypti*, *Aedes vexans*, *Anopheles gambiae*, and *Culex quinquefasciatus* [64, 65, 66, 67, 68, 69]. The antiviral role of piRNAs in mosquitoes has previously been reviewed by other authors [70].

### Lepidoptera

In the silkworm *B. mori*, piRNAs are also present in both germline and somatic cells of ovaries and testes, where they are mostly associated with the repression of retrotransposons [71, 72, 73, 74]. In addition, piRNAs are also expressed during embryogenesis and in other non‐gonadal tissues such as in the fat body and midgut, where it has been hypothesized that they play a role against viral infections [75]. A recent study has also shown that non‐gonadal somatic piRNA expression suppresses transposon activity and maintains the normal expression of host genes [76].

Interestingly, the most remarkable role of piRNAs in the silkworm is their role in sex determination during embryogenesis. A piRNA called *Fem*, which is expressed during female embryogenesis, maintains the female‐specific *doublesex* splicing pattern by repressing the expression of the *Masc* gene, which is involved in the silkworm sex determination cascade [74, 77]. Regulation of the *Masc* gene has also been observed in the gonads, where it controls sex‐specific gene expression [77]. In knockout individuals of the *Gtsf1* gene, which is involved in the cleaving activity of *Siwi* (the silkworm counterpart of *aub*) developmental abnormalities have been reported in both testes and ovaries [78, 79].

The three Lepidoptera species studied by Lewis *et al*. [16], the moths *Trichoplusia ni* and *Plutella xylostella*, and the butterfly *Heliconius melpomene*, all display piRNA expression in both germline and thoracic tissues. Independent studies in other moths also reported abundant non‐gonadal piRNA expression. In *Manduca sexta*, piRNAs are expressed in the muscle where reportedly they play a role in cell death control [80], and in the *Helicoverpa armigera*, piRNAs were found abundantly in the fat body, midgut, and central nervous system [81].

### Hymenoptera

Although transposon expression in honeybees is low, the biogenesis pathways and functions of piRNAs appear to be conserved [82, 83]. In the honeybee *A. mellifera*, piRNA expression has been observed in ovaries and testes, as well as in non‐gonadal somatic tissues such as in male and female thorax [16]. In addition, piRNAs in this species are present in fertilized and unfertilized eggs, and they potentially target sex‐determination loci during embryogenesis [82, 83].

In the bumblebee *Bombus terrestris*, piRNA expression was detected in the testes and ovaries, but not in the thorax [16]. In the ant species *Temnothorax rugatulus*, somatic piRNA expression was detected in the brain and thorax, but at a much lower level than in the ovaries, and virtually no ping‐pong signatures were detected in the brain [84].

### Coleoptera

In the red flour beetle *T. castaneum*, piRNAs are maternally loaded into the oocytes and control TE activity during embryogenesis [85]. Moreover, in *T. castaneum* and another beetle, the corn rootworm *Diabrotica virgifera*, piRNAs have also been reported to be expressed in both gonads and thoracic tissues [16]. This contrasts with another beetle, the burying beetle *Nicrophorus vespilloides*, in which the same authors only reported the detection of piRNAs in the gonads [16].

### Hemiptera

The genome of the pea aphid *A. pisum* contains the largest reported number of *piwi* and *AGO3* genes, with at least eight *piwi* genes and two *AGO3* genes [86]. Expression of *piwi* and *AGO3* genes was detected in both germ cells and somatic cells of the gonads, with some genes being expressed exclusively in one cell type. In addition, notable variation in expression levels across reproductive morphs was observed [86]. Abundant piRNA expression was also detected in the thoracic tissue of this species [16].

While *A. pisum* is arguably the most well‐studied hemipteran species, piRNAs have also been studied in other hemipteran species. In another aphid, *Aphis gossypii*, specimens fed on a melon strain containing a resistant gene against this aphid infection showed an increase of the 27nts RNAs [87]. This RNA length coincides with the length of piRNAs, and led to the proposal that piRNAs could be important factors for the ability of aphids to feed on a given host, and for the development of plants with resistant genes to aphids [87].

Among non‐aphid hemipteran species, the psyllid *Diaphorina citri* has been reported to produce endogenous endovirus‐derived piRNAs in different tissues including the head, gut, and hemolymph. In the tobacco whitefly *Bemisia tabaci*, expression of genes involved in the piRNA pathway has been detected in the gut and salivary glands, as well as in the whole body of adults [88]. In the soybean pest *Riptortus pedestris*, abundant non‐gonadal piRNAs have recently been detected and investigated as potential novel biopesticides [89]. In the Chagas disease vector, the kissing bug *Rhodnius prolixus*, piRNAs have been described in the somatic follicular cells of the ovaries [90], and more recently in the embryo, where they target horizontally transferred transposons derived from the vertebrates on which *R. prolixus* feeds [91].

### Blattodea

In the cockroach *B. germanica*, piRNA expression from both primary and secondary pathways [92] was detected in 22 whole‐body small RNA‐seq libraries from 11 stages of embryonic and post‐embryonic development [93]. Both piRNA pathways appeared to be active at all stages, with distinct piRNA clusters showing stage‐specific expression profiles [92]. In the same species, piRNAs were shown to be involved in the control of the short interspersed nuclear elements (SINEs) [94].

Termites are the sister group of cockroaches and common models to study eusociality with phenotypically and behaviorally differentiated morphs [95]. In the highly social termite *Macrotermes bellicosus*, genes related to piRNA biogenesis, including ping‐pong factors, were found to be highly expressed in brain transcriptomes [96]. Interestingly, the authors found several of those genes, including *aub1*, *qin*, and *zuc* highly abundant in transcriptomes from heads. Furthermore, these genes were differentially down‐regulated in the old short‐living major workers compared to young ones, while the same piRNA‐related genes were not down‐regulated in old long‐living reproductive queens compared to young ones. These observations suggested that piRNAs might be active in the termite's brain as an anti‐aging mechanism. This hypothesis could explain the long life of queens, which is up to 20 years, compared to workers who live only a few months and have higher TE activity in the brain [96].

### Orthoptera

In the Mediterranean field cricket *G. bimaculatus*, small RNA‐seq reads of piRNA length (27–29 nts) were found in brains with a similar abundance to reads of miRNA length (21–22 nts), suggesting that piRNAs might be highly expressed in the cricket brain [97]. Furthermore, the *piwi* gene is expressed in the brain's mushroom body neuroblasts and is involved in the formation of long‐term memory [97]. Despite the involvement of PIWI in long‐term memory formation in *G. bimaculatus* and the possible high expression of piRNAs in its brain, the direct involvement of piRNAs in long‐term memory formation in crickets has not been demonstrated yet.

In addition, the *G. bimaculatus* genome appears to have undergone expansion of the main genes involved in piRNA biogenesis, having three *AGO3* and four *piwi* genes [43, 97]. The expression of these genes was detected in transcriptomes of male and female gonads, brains, ventral cords, and carcasses.

A recent study on *Locusta migratoria* showed that piRNAs are expressed in its brain and can regulate food intake in this locust species [98]. In the same species, in 2009, in one of the first studies using small RNA‐seq in hemimetabolous insects, the authors found a significant difference in the 26–29 nts RNAs between solitary and gregarious phases [99]. These results may indicate abundant expression of non‐gonadal piRNAs, and a role in changing between solitary and gregarious phases of this species.

## Non‐gonadal expression of piRNAs in other arthropods

While most of the genetic data from arthropods comes from insects, non‐gonadal somatic piRNA has also been described in a few other arthropod species.

Lewis *et al*. [16] reported the detection of piRNA expression in the soma and germline of the horseshoe crab *Limulus polyphemus*, the scorpion *Centruroides sculpturatus*, the spider *Parasteatoda tepidariorum*, and the centipede *Strigamia maritima*. The only one, from the investigated non‐insect arthropod species, in which the authors did not detect piRNA expression in somatic tissue was the crustacean pill bug *Armadillidium vulgare*, which displayed abundant piRNA expression only in male and female gonads. In another crustacean, the planktonic *Daphnia magna*, piRNA expression was detected in embryos and whole‐body adults (including ovaries), in which piRNAs displayed age‐related expression patterns [100]. In other crustaceans, research on piRNAs and related factors has been mainly restricted to the gonads, such as in the mud crab *Scylla paramamosain* [101] and the Asian tiger prawn *Penaeus monodon* [102].

In the two‐spotted spider mite *Tetranychus urticae*, piRNA expression was detected in small RNA‐seq from whole bodies of adults, nymphs and larvae, and embryos, but only experimentally validated in gonads. Interestingly, this species has putatively lost *Zucchini*, which, the authors suggest, may have been replaced by a siRNA‐mediated activity [103].

## Overview of the literature reporting non‐gonadal piRNA expression in Arthropoda

In our extensive literature research, we identified reports of gonadal piRNA expression in virtually all arthropodan species in which piRNAs have been studied. The presence of abundant ping‐pong piRNAs during embryogenesis is also widely reported across arthropod lineages. Furthermore, we showed that in recent years, non‐gonadal piRNA expression has been reported in a wide range of arthropod species, including insects, myriapods, crustaceans, and chelicerates (Table S1).

In mammals, it has been argued that a large fraction of the non‐gonadal somatic expression of piRNAs may be an artifact derived from the misannotation of other ncRNAs as piRNAs [104]. Nevertheless, this is unlikely in arthropods, given the large and growing body of literature reviewed above on non‐gonadal piRNA expression across multiple different species. The majority of this literature comprises individual articles reporting non‐gonadal piRNA expression in a particular species, often the main research organism of the corresponding laboratory. This means that, except for Lewis *et al*. [16], most of the available information is derived from disparate reports, rather than through comprehensive piRNA analysis across species with a standardized methodology that would allow direct comparisons between species.

Hence, here we performed a large‐scale analysis of publicly available small RNA‐seq datasets across 17 arthropod species with a standardized and robust methodology. Moreover, we include data from different tissue types to gain additional insights into which tissues typically express piRNAs in each lineage.

## Publicly available small RNA‐seq confirms widespread non‐gonadal piRNA expression across arthropods

### Data retrieval and analysis

To obtain datasets in which we could ascertain the presence of piRNA expression, we queried the NCBI database for all arthropod species with a reference genome, and for each of the selected species, we obtained the metadata of all its RNA‐seq datasets. Using this metadata, we selected datasets corresponding to small RNA‐seq, and subsequently classified them into the following categories: male or female reproductive tissue (which included gonads, sperm, and oocytes), embryo, whole body, nervous system, and other somatic tissues (which included, gut, fat body, midgut, gill, epidermis, antennae, thorax, muscle, and legs); the unclassified datasets were discarded.

**Fig. 3:**
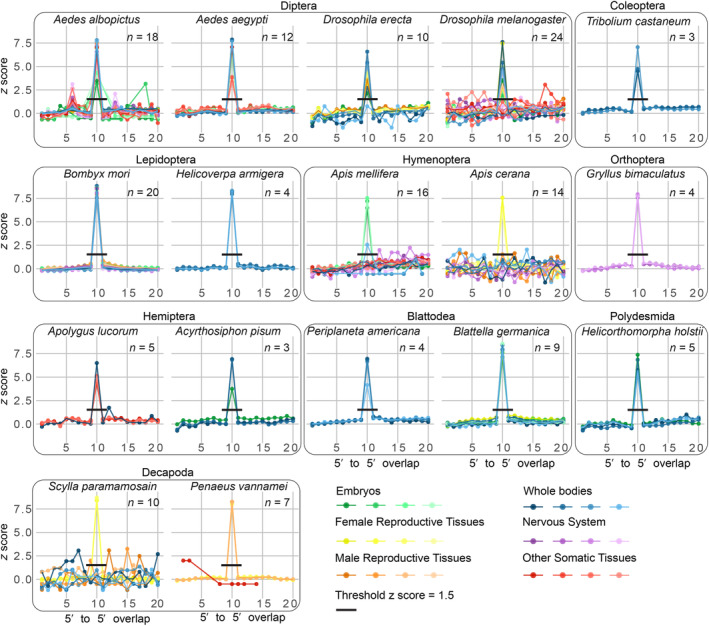
piRNA ping‐pong signatures across samples. The number of 5′‐to‐5′ overlaps (*z*‐score transformed) from zero to 20nts between expressed loci in small RNA‐seq data for each of the 17 analyzed species. Each small RNA‐seq sample is colored by tissue type, and different samples from the same tissue are plotted with a distinct shade of the same color. The peak at 10nts overlap corresponds to the ping‐pong signature, if the 10nts is the highest peak and with a *z*‐score > 1.5 (black line), we consider it evidence of piRNA expression in the given sample.

Then, whenever possible, we selected two species per order for which we had at least one small RNA‐seq dataset from both somatic and reproductive tissues. For orders in which multiple species were eligible, we picked those with a more complete set of data. Given the large amount of data from Diptera, we included two flies and two mosquitoes. To include representatives from the major orders in which no species had enough data, we additionally added species for which there was no eligible reproductive tissue data (detailed methods in Data S1). The 168 small RNA‐seq datasets selected (Table S2) were processed as described in Data S1, and the 5′‐to‐5′ overlaps between cleaned mapped reads were calculated. The presence of the ping‐pong signature was determined by the observation of a 5′‐to‐5′ peak at 10 nucleotides with a *z*‐score > 1.5 (Fig. 3). The presence of a ping‐pong signature was considered robust evidence of piRNA expression in a given dataset. It should be noted that the lack of ping‐pong signatures does not suggest a lack of piRNA expression, since primary piRNAs could still be expressed.

### Detection of ping‐pong signature in small RNA‐seq datasets from Arthropoda

**Fig. 4:**
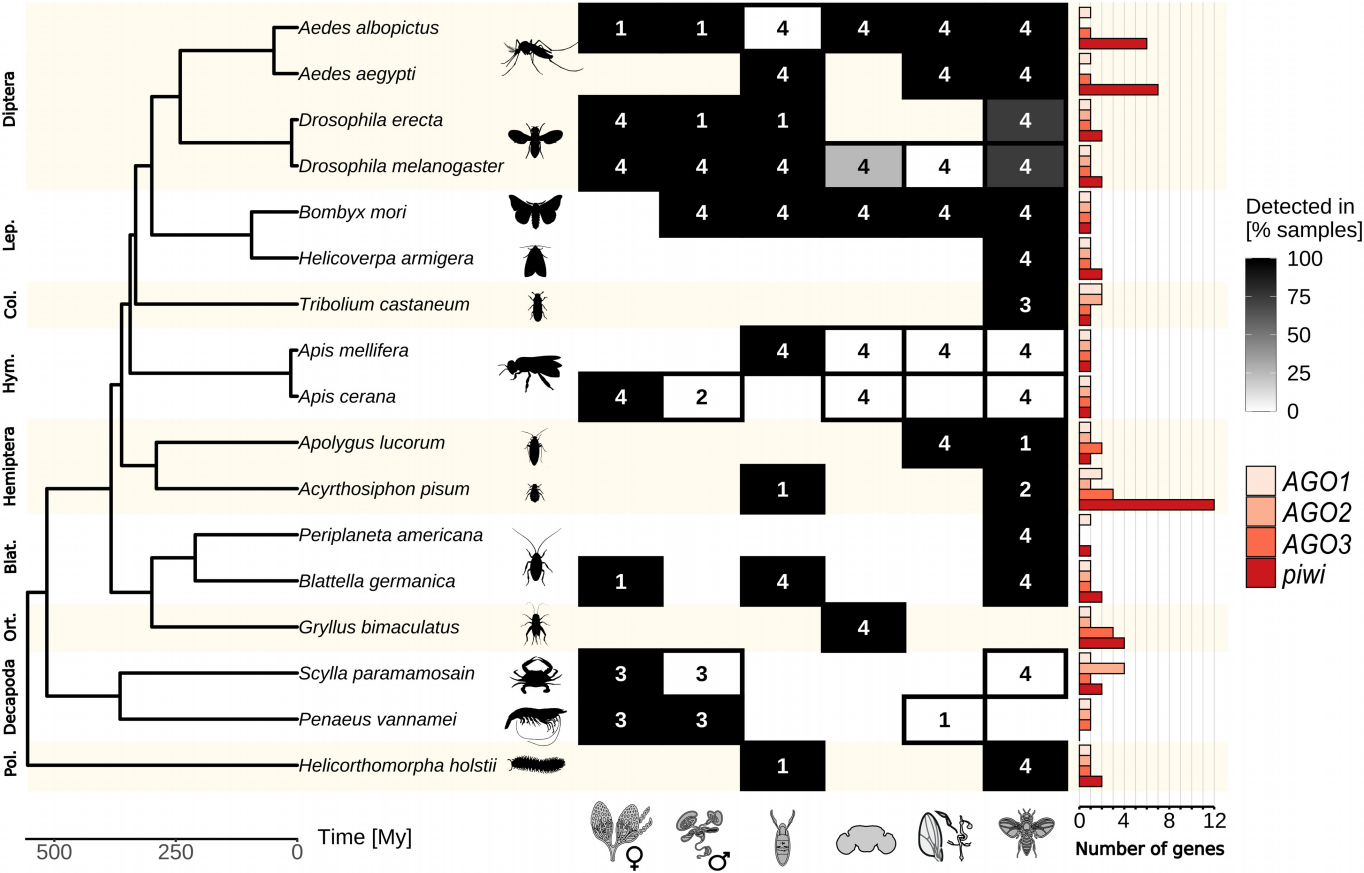
Ping‐pong piRNA expression per tissue in each species. The species included in our analysis are shown in the phylogenetic tree, with the number of small RNA‐seq samples analyzed for each tissue type (column) in the boxes (lack of a box means no samples analyzed). The box fill gradient indicates the percentage of samples in which we detected the ping‐pong piRNA signature in the given species and tissue type. From left to right, the columns represent samples from female reproductive tissue, male reproductive tissue, egg/embryo, nervous system, other somatic tissues, and the whole body. Bar charts indicate the number of potential *AGO1*, *AGO2*, *AGO3*, and *piwi* genes in each species. The representative tissue silhouettes are from bioicons.com, species silhouettes from phylopic.org, and the phylogenetic tree was obtained from TimeTree [106].

The results of this analysis confirmed the widespread presence of ping‐pong piRNA expression in various non‐gonadal tissues in all analyzed distant arthropod orders separated by over 500 million years (Fig. 4).

As expected, in female reproductive tissues, ping‐pong piRNA expression was detected in all samples from all species studied. Similarly, in male reproductive tissues, ping‐pong piRNAs were detected in all samples, with the remarkable exceptions of the samples from the bee *Apis cerana* and the crab *S. paramamosain*. In embryos, we detected ping‐pong signatures in all processed samples except in all four samples from the mosquito *Ae. albopictus*.

In *D. melanogaster*, interestingly, we detected ping‐pong signatures in gonads and embryos, but very few in other tissues. Specifically, within the nervous system tissue category, we detected ping‐pong piRNA in one sample from the brain, while we did not detect it in the other three samples from whole heads. Exploration of the specific brain sample in which we identified ping‐pong piRNA expression, revealed that the detection of ping‐pong comes from only 27 ping‐pong loci, a very low number compared to the thousands of ping‐pong loci we found in ovarian samples. In the other somatic tissue category, we did not detect ping‐pong piRNAs in any of the samples, which included muscle and midgut tissues. As for the whole‐body samples, the ping‐pong signature was present and abundant in three out of the four samples, with the number of ping‐pong loci ranging from 100 to 1900 per sample. Given that whole bodies contain gonads, we cannot rule out the possibility that ping‐pong piRNAs came from the gonads. But considering that gonads account for only a small portion of the whole body, it seems unlikely that the detection of significant ping‐pong piRNA signature could come exclusively from gonads, and supports the observation that there might be other tissues, like the nervous system, which might also produce ping‐pong piRNAs. In the other *Drosophila* species included in the analysis, *Drosophila erecta*, ping‐pong piRNAs were also present in all reproductive tissue and embryo samples, and in three of four whole‐body samples.

Altogether, both the literature review and our analyses suggest that some *Drosophila* species, including *D. melanogaster*, with a much reduced piRNA production in non‐gonadal tissues, might be exceptional even among flies.

In bees, represented in our analyses by *A. mellifera* and *A. cerana*, we detected ping‐pong piRNAs in all female reproductive tissue and embryo samples, but not in male reproductive tissue. As for non‐gonadal and non‐embryonic tissues, we did not detect ping‐pong piRNAs in any of the eight whole‐body samples, eight nervous system samples, or four gut samples. Lewis *et al*. [16] found piRNA expression in the thorax of *A. mellifera*, but did not detect it in the thorax of another Hymenoptera, the bumblebee *B. terrestris*. These results suggest that non‐gonadal non‐embryonic ping‐pong piRNA expression might occur in bees and bumblebees, but in a more restricted set of tissues or stages than in most other insects.

In the two crustaceans analyzed, the crab *S. paramamosain* and the shrimp *Penaeus vannamei*, ping‐pong piRNAs were not detected in any of the five non‐gonadal non‐embryonic tissue categories (Fig. 4). This is congruent with the reported lack of somatic piRNA expression in the studied terrestrial crustacean *A. vulgare* [16]. In addition, in our literature review, we also failed to identify any report of piRNA expression in non‐embryonic non‐gonadal tissue in any crustacean, with the described putative exception of the planktonic *D. magna* [100]. Further studies including more crustacean species and more tissues are needed to confirm the hypothesis that piRNA expression in Crustacea may be restricted to gonads and embryos.

The current data point to the further hypothesis that the lack of abundant non‐gonadal non‐embryonic piRNA expression in Crustacea does not represent the ancestral trait, but rather a loss of this feature. This hypothesis is supported by the non‐gonadal piRNA expression in early branching arthropod species, such as in the herein‐analyzed myriapod *Helicorthomorpha holstii* (Fig. 4), as well as in the Lewis *et al*. [16] analyzed myriapod *Strigamia maritima*, and the chelicerates *L. polyphemus*, *C. sculpturatus*, and *P. tepidariorum*.

## *Argonaute* gene copy evolution

The *Argonaute* gene superfamily is subdivided into the *AGO1*, *AGO2*, *AGO3*, and *piwi* gene subfamilies. *AGO1* and *AGO2* genes are involved in miRNA and siRNA pathways, respectively [105], while *AGO3* and *piwi* genes belong to piRNA pathways. Indeed, *piwi* (and its paralogue in flies *aub*) and *AGO3* proteins are the proteins that directly bind to piRNAs. Given the important roles of these genes in piRNA production, we wondered whether to achieve higher tissue diversity of piRNA expression, a larger repertoire of *AGO3* and *piwi* genes could be required.

For this purpose, for each of the 17 species analyzed here, we automatically identified *Argounate* genes based on the presence of consecutive PAZ and Piwi domains in the protein sequence. Then, we subdivided them into *AGO1*, *AGO2*, *AGO3*, and *piwi* based on the clustering on the gene tree (Data S1; Fig. S1).

Our results showed few variations in *AGO1* and *AGO2* gene numbers, with 15 species having a single *AGO1* and 12 species having a single *AGO2* (Fig. 4; Table S3). In contrast, the number of *piwi* and *AGO3* genes, which are the subfamilies involved in the piRNA pathways, is more variable.

In the case of *piwi*, we identified six species with a single *piwi* gene, six species with two *piwi* genes, *G. bimaculatus* with four *piwi* genes as previously described [97], the two mosquitoes with six and seven *piwi* genes, and *A. pisum* with 12 *piwi* genes. As for *AGO3*, most species (13) have a single *AGO3* gene. The exceptions are *Apolygus lucorum* with two *AGO3*, and the previously reported expansions in *A. pisum* and *G. bimaculatus* [86, 97] with three *AGO3* genes each. Despite using the transcript sequences obtained from the published genome annotations, it is possible that the third *AGO3* of *A. pisum*, as well as four of the 12 *piwi genes* of this species, could be the previously reported pseudogenes [86].

Overall, this analysis suggests that there is no relationship between the number of *piwi* and *AGO3* genes, and the number of tissues in which piRNA expression occurs. Therefore, it is likely that the proteins that bind to piRNAs in the gonads are the same as those that act in non‐gonadal tissue. This analysis also highlights the differences between the miRNA and siRNA pathways compared to the piRNA pathway. While, despite some exceptions, the miRNA and siRNA pathways have a very stable number of *AGO1* and *AGO2* genes respectively, piRNAs pathway has a more variable number of *AGO3* and *piwi* genes.

## Closing remarks

- Dozens of independent studies, each focusing on different species, show non‐gonadal expression of piRNAs. Taken together, it becomes clear that non‐gonadal piRNA expression is common across arthropod lineages.
- Our analysis of small RNA‐seq data across arthropods further supports this new paradigm of widespread non‐gonadal expression of piRNAs in Arthropoda.
- Current data suggest that Crustacea might have lost piRNA expression beyond gonads and embryos. However, data from this lineage is scarce, and further studies are required to test this hypothesis.
- Even in *D. melanogaster*, for a long time thought to restrict piRNA expression to the gonads, current evidence suggests that it also expresses piRNAs in non‐gonadal tissue. However, unlike most of the other arthropods analyzed, ping‐pong piRNAs in non‐gonadal tissues are often found at very low levels.
- Some prevalent views on piRNAs in arthropods are highly influenced by the observation in the genetic model *D. melanogaster*. Here, we show that broadening the research to other experimental organisms provides a better understanding of piRNA biology and highlights the benefits of research in non‐model organisms.
- All these observations open the door to further questions such as: Are the non‐gonadal piRNAs produced by the same biogenesis pathways? Are the same proteins involved in their biogenesis? What are the roles of somatic piRNAs? Could they play general roles in regulating mRNA expression?

## Author contributions

TY: Initial literature review and writing the first version of the draft and figures. KK: Data analysis. RM, DR and SS: Development of computational pipelines and data processing. TG and KK: Data visualization. GY: Conceptualization, supervision, literature review, and writing the final version of the manuscript. All authors reviewed and approved the manuscript.

## Supporting information


**Fig. S1.** Maximum likelihood tree of the Argonaute‐family proteins obtained from the genome annotations of the 17 species that contained PAZ (PF02170) and Piwi (PF02171) domains.
**Table S1.** Summary of arthropod orders discussed in this manuscript for which we found reports of non‐gonadal piRNA expression.
**Table S2.** List of the 168 datasets analyzed, with their run accession identifier from NCBI‐SRA, species name, tissue type to which we assigned the sample, and whether or not we identified ping‐pong signature in that sample.
**Table S3.** Number of genes belonging to each of the four *Argonaute* sub‐families, *AGO1*, *AGO2*, *AGO3*, and *piwi* (including flies *aub*) identified in each species genome annotation.
**Data S1.** Supplementary Methods.

## Data Availability

All data analyzed in this article were obtained from NCBI. The accession identifiers of all analyzed datasets are available in Table S2.
