# Supplementary material for: Non‐gonadal expression of piRNAs is widespread across Arthropoda
Source: FEBS Lett. 2024 Oct 2;599(1):3–18. doi: 10.1002/1873-3468.15023 (PMC11726155; doi:10.1002/1873-3468.15023)
Supplement: Supplementary file 1 — Fig. S1. Maximum likelihood tree of the Argonaute‐family proteins obtained from the genome annotations of the 17 species that contained PAZ (PF02170) and Piwi (PF02171) domains. Table S1. Summary of arthropod orders discussed in this manuscript for which we found reports of non‐gonadal piRNA expression. Table S2. List of the 168 datasets analyzed, with their run accession identifier from NCBI‐SRA, species name, tissue type to which we assigned the sample, and whether or not we identified ping‐pong signature in that sample. Table S3. Number of genes belonging to each of the four Argonaute sub‐families, AGO1, AGO2, AGO3, and piwi (including flies aub) identified in each species genome annotation. Data S1. Supplementary Methods. [file FEB2-599-3-s001.docx]

**Supplementary Information**

**Non-gonadal expression of piRNAs is widespread across Arthropoda**

Takahisa Yamashita*^1^, Krystian Komenda*^1,2^, Rafal Milodrowski^1,2^, Dominik Robak^1^, Szymon Szrajer^1^, Tomasz Gaczorek^1^, Guillem Ylla^1^

^1^ Laboratory of Bioinformatics and Genome Biology, Faculty of Biochemistry, Biophysics and Biotechnology, Jagiellonian University, Krakow, Poland

^2^ Jagiellonian University, Doctoral School of Exact and Natural Sciences, Jagiellonian University, Krakow, Poland

* Contributed equally

Contents

[Supplementary Methods 1](#_Toc165024151)

[Small RNA-seq data retrieval 1](#_Toc165024152)

[Small RNA-seq data analysis 2](#_Toc165024153)

[Identification of Argonaute family genes 3](#_Toc165024154)

[Supplementary Figures 4](#_Toc165024155)

[Supplementary Figure S1 4](#_Toc165024156)

[Supplementary Tables 5](#_Toc165024157)

[Supplementary Table S1 5](#_Toc165024158)

[Supplementary Table S2 6](#_Toc165024159)

[Supplementary References 7](#_Toc165024160)

# **Supplementary** Methods

## Small RNA-seq data retrieval

We identified all the arthropods with available genome assembly in NCBI. For each of the identified species, we obtained the metadata of all its RNA-seq datasets available in NCBI. Using the metadata provided by the submitters of the RNA-seq, we filtered those corresponding to small RNA-seq as follows. First, we removed all datasets that had "LibrarySource" other than "TRANSCRIPTOMIC". Second, we retained only samples with Instrument" being BGISEQ, DNBSEQ, NextSeq, HiSeq, Torrent, and MGISEQ. Third, we selected samples that had "Assay Type" as "miRNA-Seq" and "AvgSpotLen" smaller or equal than 200, or "Assay Type" as "RNA-Seq" and "AvgSpotLen" smaller or equal than 60.

Subsequently, we selected those datasets that could fit into one of the following tissue categories: male or female reproductive tissue, embryo, whole body, nervous system, other somatic tissues (gut, fat body, midgut, gill, epidermis, antennae, thorax, muscle, and leg ). This classification was done as follows:

First, we classified samples as "Other_somatic_tissues" if the metadata column "tissue" contained: "gut", "midgut", "fatbody", "fat body", "gill", "edpidermis", "antennae", "thorax", "muscle", "legs".  The samples were then classified as "Whole_body" if the metadata column "tissue" contained: "whole body", "wholebody", "whole insect", "body", "whole animal", "whole organism", 'whole fly", "whole mosquitoes", "whole mosquito", "whole bee", "insect", "whole", "entire bodies", "the whole body". Samples were assigned to "Nervous_system" if the metadata column "tissue" contained: "brain", "head", "whole heads", "heads", "brains". Samples were assigned to "Female_Reproductive_Tissue" if the metadata column "tissue" contained: "ovary", "ovary", "ovarioles", "oocyte", "female germline", "female reproductive tract". Samples were assigned to "Male_Reproductive_Tissue" if the metadata column "tissue" contained: "testes", "testis", "sperm". Lastly, samples were assigned to "Embryo" if in the meetada columns "Developmental_stage", "dev_stage", "Age" or "tissue" contained "embryonic", "embryo", "embrios", "embryos", "whole embryo", or "egg".

Then, whenever possible, we selected two species per order for which we had at least a small RNA-seq from a gonadal and a non-gonadal tissue. For orders in which multiple species were eligible, we picked those with more tissues types sequenced. For Diptera we selected as representatives two species of flies and two of mosquitoes, due to the large number of datasets for this order. To include representatives from most major orders, for some orders in which no species had enough data, we included species in our analysis despite the lack of eligible gonadal tissue data. The final list of analyzed datasets is shown in **Supplementary Table S1.**

## Small RNA-seq data analysis

For each of the selected species above, we randomly selected up to four datasets for each tissue category. With the SRA-toolkit v2.11.3, we downloaded the forward reads of the RNA-seq data, then trimmed adapters with TrimGalore v0.6.7 (<https://github.com/FelixKrueger/TrimGalore>), and used FastQC v0.11.9 [1] to check for quality control. Then we mapped the RNA-seq data to the reference genome with Bowtie2 v2.4.4 [2], and only samples with >49% of aligned reads to the reference genome were retained. Discarded samples due to a low percentage of mapped reads were replaced by another sample from the same tissue category if available.

To avoid inflating the number of ping-pong signatures reads mapped in the same genome locus with extremes varying in length up to 3nts, were considered as the same expressed locus. Then, we calculated the 5’-to-5’ overlaps between all expressed loci. To make the number of overlaps comparable between samples with different sample sizes, we applied a z-score transformation and represented them graphically (**Figure 3**). The presence of unequivocal ping-pong piRNA was determined by the presence of the highest 5'-to-5' peak at 10nt overlap with a z-score > 1.5.

## Identification of Argonaute family genes

We extracted the aminoacid sequences of all annotated genes of each of the 17 species using the genome assembly and annotation files with gffread v0.12.7 [3]. In the aminoacid sequences, we search for the presence of PAZ (PF02170) and Piwi (PF02171) domains (e-value cutoff 0.01) with hmmsearch v3.4 ([https://hmmer.org](https://hmmer.org/)). The protein sequence of the largest transcript of a gene that contained a consecutive presence of PAZ and Piwi domains was treated as a putative *Argonaute* gene. We aligned the putative amino acid sequence of Argonautes together with 39 previously characterized sequences of this family [4] with MUSCLE v5.1[5], and built a phylogenetic gene tree with RAxML v. 8.2.12[6] (PROTGAMMAAUTO, bootstrap 100). The known *Argonaute* genes belonging to the same subfamily (*AGO1*, *AGO2*, *AGO3*, or *piwi*) grouped together and separated from members of the other subfamilies (**Supplementary Figure S1**). Based on this, we assigned all proteins of each cluster to one of the four sub-families of *Argonaute* genes, and counted the number of each sub-family in each species (**Supplementary Table S2**).

#
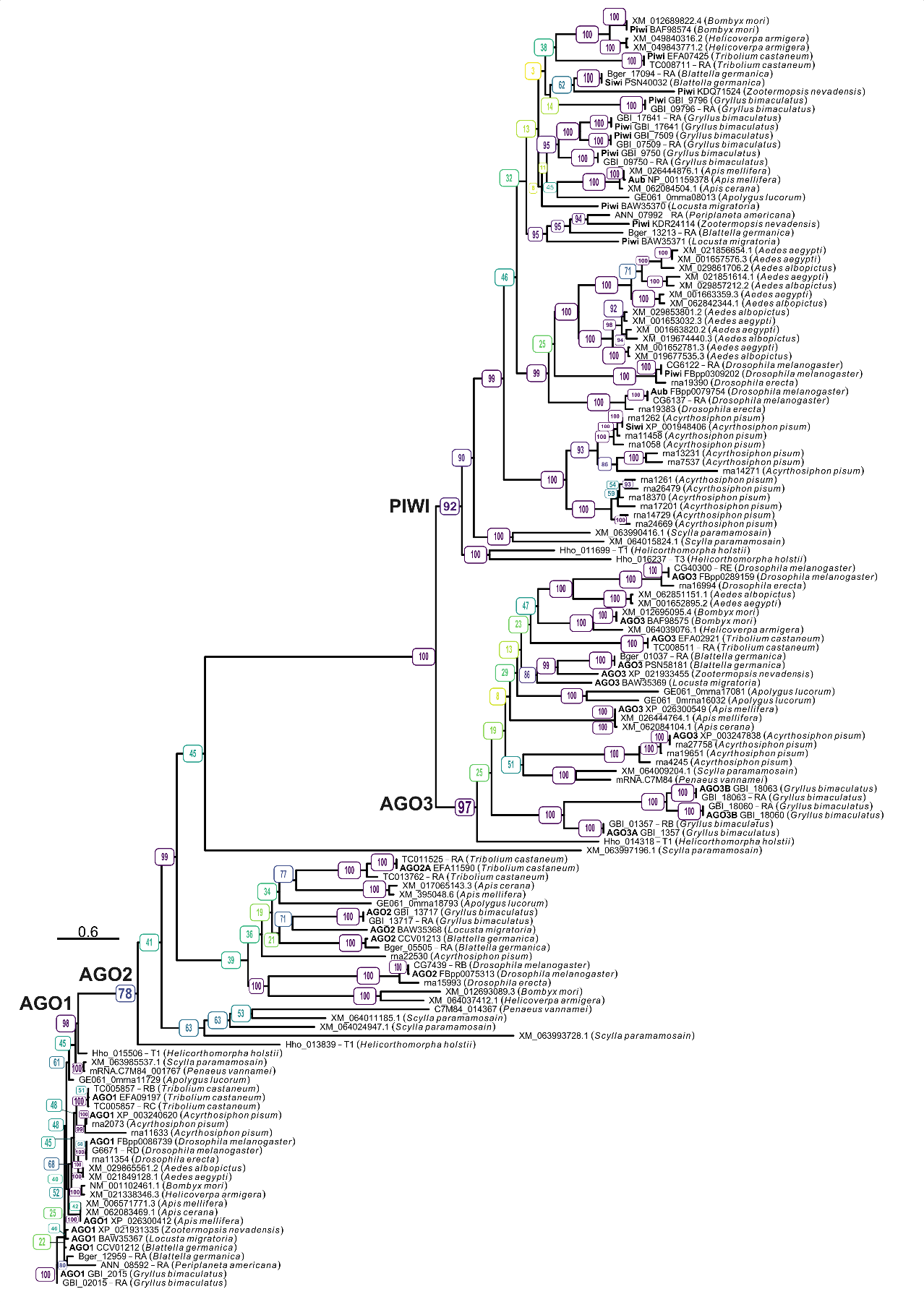
Supplementary Figures

Supplementary Figure S1**:** Maximum likelihood tree of the Argonaute-family proteins obtained from the genome annotations of the 17 species that contained PAZ (PF02170) and Piwi (PF02171) domains. The tree also includes 39 known AGO1, AGO2, AGO3, and PIWI sequences, which we use to assign nodes to these four subfamilies, and subsequently classify all proteins in each node as belonging to that sub-family. Values in the nodes indicate the bootstrap support from RAxML with 100 replicates.

# Supplementary Tables

Supplementary Table S1**:** Summary of arthropod orders discussed in this manuscript for which we found reports of non-gonadal piRNA expression. For each order, up to two representative species are shown, together with some of the relevant citations reporting the piRNA detection.

| **Order** | **Representative Species** | **Gonadal tissue** | | **Non-gonadal** | |
| --- | --- | --- | --- | --- | --- |
|  |  | **Ovary** | **Testes** | **Embryo** | **Other organs** |
| **Diptera** | Flies (*Drosophila melanogaster*) | (Brennecke et al, 2007a) | (Quénerch’du et al, 2016) | (Rouget et al, 2010) | (Yan et al, 2011) Head Imaginal discs  (Perrat et al, 2013) Brain |
|  | Mosquitos (*Aedes aegypti*) | (Gamez *et al*, 2020) | | (Halbach et al, 2020), (Betting et al, 2021) | (Gamez et al, 2020) Larva  (Lewis et al, 2018) Thorax |
| **Lepidoptera** | Silk worms (*Bombyx mori*) | (Kawaoka et al, 2008) | (Kiuchi et al, 2023) | (Kiuchi et al, 2014b) | (Kiuchi et al, 2023)　Imaginal discs,  (Verdonckt et al, 2023) Midgut, Fatbody |
|  | Moths (*Manduca sexta*) | (Lewis et al,2018) | | | (Tsuji et al, 2021) Muscle |
| **Hymenoptera** | Bees (*Apis mellifera*) | (Watson *et al*, 2022) | | (Wang et al, 2017) | (Xu et al, 2022), Thorax |
|  | Ants (Temnothorax rugatulus) | (Seistrup et al, 2023) |  |  | (Seistrup et al, 2023) Brain |
| **Coleoptera** | Beetles (*Tribolium castaneum*) | (Lewis et al,2018) |  | (Ninova et al, 2017) | (Lewis et al,2018) Thorax |
| **Hemiptera** | Aphids (*Acyrthosiphon pisum*) | (Lu et al, 2011) | | | (Lewis et al,2018) Thorax |
|  | Lices (*Bemisia tabaci*) |  |  |  | (Mondal et al, 2020) Gut, Salivary glands, Whole body |
|  | Chagases (*Rhodnius prolixus*) | (Brito et al, 2018) |  | (Brito et al, 2024) |  |
| **Blattodea** | Cockroaches (*Blattela germanica*) | (Ylla et al, 2017) (Llonga et al,2018) | | | (Ylla et al, 2017) Whole body  (Llonga et al,2018) Whole body |
| **Orthoptera** | Crickets (*Gryllus bimaculatus*) |  |  |  | (Kulkarni et al, 2023) Brain |
|  | Locusts (*Locusta migratoria*) |  |  |  | (Wang et al, 2022) Brain (Wei et al, 2009) |
| **Other arthropods** | Crabs (*Limulus polyphemus*) | (Lewis et al,2018) | |  | (Lewis et al,2018) Thorax |
|  | Scorpions (*Centruroides sculpturatus*) |  |  |  |  |
|  | Spiders (*Parasteatoda tepidariorum*) |  |  |  |  |
|  | Centipedes (*Strigamia maritima*) |  |  |  |  |
|  | Water Fleas (*Daphnia magna*) | (Hearn et al, 2022) |  | (Hearn et al, 2022) | (Hearn et al, 2022) Whole body |
|  | Prawns (*Penaeus monodon*) |  | (Sukthaworn et al, 2020) |  |  |

Supplementary Table S2**:** List of the 168 datasets analyzed, with their run accession identifier fromNCBI-SRA, species name, tissue type to which we assigned the sample, and whether or not we identified ping-pong signature in that sample.

| **Accession** | **Species** | **Tissue type assigned** | **Ping-pong** |  | **Accession** | **Species** | **Tissue type assigned** | **Ping-pong** |
| --- | --- | --- | --- | --- | --- | --- | --- | --- |
| SRR14670936 | *Acyrthosiphon pisum* | Embryo | TRUE |  | SRR14126108 | *Apis cerana* | Female_Reproductive_Tissue | TRUE |
| SRR17183776 | *Acyrthosiphon pisum* | Whole_body | TRUE |  | SRR14126095 | *Apis cerana* | Male_Reproductive_Tissue | FALSE |
| SRR6981552 | *Acyrthosiphon pisum* | Whole_body | TRUE |  | SRR14126096 | *Apis cerana* | Male_Reproductive_Tissue | FALSE |
| SRR13810515 | *Aedes aegypti* | Embryo | TRUE |  | SRR14126087 | *Apis cerana* | Nervous_system | FALSE |
| SRR13810516 | *Aedes aegypti* | Embryo | TRUE |  | SRR14126088 | *Apis cerana* | Nervous_system | FALSE |
| SRR13810517 | *Aedes aegypti* | Embryo | TRUE |  | SRR14126097 | *Apis cerana* | Nervous_system | FALSE |
| SRR13810522 | *Aedes aegypti* | Embryo | TRUE |  | SRR14126098 | *Apis cerana* | Nervous_system | FALSE |
| SRR11870698 | *Aedes aegypti* | Other_somatic_tissues | TRUE |  | SRR13959549 | *Apis cerana* | Whole_body | FALSE |
| SRR6123581 | *Aedes aegypti* | Other_somatic_tissues | TRUE |  | SRR13959550 | *Apis cerana* | Whole_body | FALSE |
| SRR6123582 | *Aedes aegypti* | Other_somatic_tissues | TRUE |  | SRR13959570 | *Apis cerana* | Whole_body | FALSE |
| SRR6123584 | *Aedes aegypti* | Other_somatic_tissues | TRUE |  | SRR13959571 | *Apis cerana* | Whole_body | FALSE |
| SRR5811303 | *Aedes aegypti* | Whole_body | TRUE |  | SRR3708803 | *Apis mellifera* | Embryo | TRUE |
| SRR5811304 | *Aedes aegypti* | Whole_body | TRUE |  | SRR5188645 | *Apis mellifera* | Embryo | TRUE |
| SRR5811305 | *Aedes aegypti* | Whole_body | TRUE |  | SRR5188647 | *Apis mellifera* | Embryo | TRUE |
| SRR5811306 | *Aedes aegypti* | Whole_body | TRUE |  | SRR5188648 | *Apis mellifera* | Embryo | TRUE |
| SRR5168326 | *Aedes albopictus* | Embryo | FALSE |  | SRR14320036 | *Apis mellifera* | Nervous_system | FALSE |
| SRR5168328 | *Aedes albopictus* | Embryo | FALSE |  | SRR14320037 | *Apis mellifera* | Nervous_system | FALSE |
| SRR5168330 | *Aedes albopictus* | Embryo | FALSE |  | SRR14320040 | *Apis mellifera* | Nervous_system | FALSE |
| SRR5168331 | *Aedes albopictus* | Embryo | FALSE |  | SRR9953274 | *Apis mellifera* | Nervous_system | FALSE |
| SRR11252299 | *Aedes albopictus* | Female_Reproductive_Tissue | TRUE |  | SRR13959554 | *Apis mellifera* | Whole_body | FALSE |
| SRR11252300 | *Aedes albopictus* | Male_Reproductive_Tissue | TRUE |  | SRR13959556 | *Apis mellifera* | Whole_body | FALSE |
| SRR11652146 | *Aedes albopictus* | Nervous_system | TRUE |  | SRR15198205 | *Apis mellifera* | Whole_body | FALSE |
| SRR11652148 | *Aedes albopictus* | Nervous_system | TRUE |  | SRR15198216 | *Apis mellifera* | Whole_body | FALSE |
| SRR11652159 | *Aedes albopictus* | Nervous_system | TRUE |  | SRR13565515 | *Apis mellifera* | Other_somatic_tissues | FALSE |
| SRR11652161 | *Aedes albopictus* | Nervous_system | TRUE |  | SRR13565516 | *Apis mellifera* | Other_somatic_tissues | FALSE |
| SRR11652149 | *Aedes albopictus* | Other_somatic_tissues | TRUE |  | SRR13565517 | *Apis mellifera* | Other_somatic_tissues | FALSE |
| SRR11652150 | *Aedes albopictus* | Other_somatic_tissues | TRUE |  | SRR6059165 | *Apis mellifera* | Other_somatic_tissues | FALSE |
| SRR11652151 | *Aedes albopictus* | Other_somatic_tissues | TRUE |  | SRR15990677 | *Apolygus lucorum* | Other_somatic_tissues | TRUE |
| SRR11652165 | *Aedes albopictus* | Other_somatic_tissues | TRUE |  | SRR15990678 | *Apolygus lucorum* | Other_somatic_tissues | TRUE |
| SRR20912167 | *Aedes albopictus* | Whole_body | TRUE |  | SRR15990683 | *Apolygus lucorum* | Other_somatic_tissues | TRUE |
| SRR20912168 | *Aedes albopictus* | Whole_body | TRUE |  | SRR15990685 | *Apolygus lucorum* | Other_somatic_tissues | TRUE |
| SRR5251237 | *Aedes albopictus* | Whole_body | TRUE |  | SRR23680036 | *Apolygus lucorum* | Whole_body | TRUE |
| SRR5251239 | *Aedes albopictus* | Whole_body | TRUE |  | SRR4252605 | *Blattella germanica* | Embryo | TRUE |
| SRR14126105 | *Apis cerana* | Female_Reproductive_Tissue | TRUE |  | SRR4252606 | *Blattella germanica* | Embryo | TRUE |
| SRR14126106 | *Apis cerana* | Female_Reproductive_Tissue | TRUE |  | SRR4252613 | *Blattella germanica* | Embryo | TRUE |
| SRR14126107 | *Apis cerana* | Female_Reproductive_Tissue | TRUE |  | SRR4252615 | *Blattella germanica* | Embryo | TRUE |
| SRR3668533 | *Blattella germanica* | Female_Reproductive_Tissue | TRUE |  | SRR5687203 | *Drosophila melanogaster* | Embryo | TRUE |
| SRR13347098 | *Blattella germanica* | Whole_body | TRUE |  | SRR23073407 | *Drosophila melanogaster* | Female_Reproductive_Tissue | TRUE |
| SRR3668535 | *Blattella germanica* | Whole_body | TRUE |  | SRR7945626 | *Drosophila melanogaster* | Female_Reproductive_Tissue | TRUE |
| SRR4252621 | *Blattella germanica* | Whole_body | TRUE |  | SRR12026860 | *Drosophila melanogaster* | Male_Reproductive_Tissue | TRUE |
| SRR4252626 | *Blattella germanica* | Whole_body | TRUE |  | SRR12026861 | *Drosophila melanogaster* | Male_Reproductive_Tissue | TRUE |
| SRR11794141 | *Bombyx mori* | Embryo | TRUE |  | SRR12213370 | *Drosophila melanogaster* | Male_Reproductive_Tissue | TRUE |
| SRR11794142 | *Bombyx mori* | Embryo | TRUE |  | SRR22857968 | *Drosophila melanogaster* | Male_Reproductive_Tissue | TRUE |
| SRR11794171 | *Bombyx mori* | Embryo | TRUE |  | SRR20830387 | *Drosophila melanogaster* | Nervous_system | FALSE |
| SRR14923460 | *Bombyx mori* | Embryo | TRUE |  | SRR20830398 | *Drosophila melanogaster* | Nervous_system | FALSE |
| SRR12047707 | *Bombyx mori* | Male_Reproductive_Tissue | TRUE |  | SRR7633524 | *Drosophila melanogaster* | Nervous_system | TRUE |
| SRR21968595 | *Bombyx mori* | Male_Reproductive_Tissue | TRUE |  | SRR8539594 | *Drosophila melanogaster* | Nervous_system | FALSE |
| SRR21968596 | *Bombyx mori* | Male_Reproductive_Tissue | TRUE |  | SRR12207879 | *Drosophila melanogaster* | Other_somatic_tissues | FALSE |
| SRR21968598 | *Bombyx mori* | Male_Reproductive_Tissue | TRUE |  | SRR7724428 | *Drosophila melanogaster* | Other_somatic_tissues | FALSE |
| SRR15211888 | *Bombyx mori* | Nervous_system | TRUE |  | SRR12207878 | *Drosophila melanogaster* | Other_somatic_tissues | FALSE |
| SRR15211889 | *Bombyx mori* | Nervous_system | TRUE |  | SRR11906535 | *Drosophila melanogaster* | Whole_body | TRUE |
| SRR15211892 | *Bombyx mori* | Nervous_system | TRUE |  | SRR14149931 | *Drosophila melanogaster* | Whole_body | FALSE |
| SRR15211893 | *Bombyx mori* | Nervous_system | TRUE |  | SRR14150381 | *Drosophila melanogaster* | Whole_body | TRUE |
| SRR25500193 | *Bombyx mori* | Other_somatic_tissues | TRUE |  | SRR11906542 | *Drosophila melanogaster* | Whole_body | TRUE |
| SRR25500199 | *Bombyx mori* | Other_somatic_tissues | TRUE |  | SRR3123324 | *Drosophila melanogaster* | Female_Reproductive_Tissue | TRUE |
| SRR25500202 | *Bombyx mori* | Other_somatic_tissues | TRUE |  | SRR10814928 | *Drosophila melanogaster* | Female_Reproductive_Tissue | TRUE |
| SRR15211887 | *Bombyx mori* | Whole_body | TRUE |  | SRR19174563 | *Gryllus bimaculatus* | Nervous_system | TRUE |
| SRR15211891 | *Bombyx mori* | Whole_body | TRUE |  | SRR19174564 | *Gryllus bimaculatus* | Nervous_system | TRUE |
| SRR4089847 | *Bombyx mori* | Whole_body | TRUE |  | SRR19174568 | *Gryllus bimaculatus* | Nervous_system | TRUE |
| SRR15211886 | *Bombyx mori* | Whole_body | TRUE |  | SRR19174569 | *Gryllus bimaculatus* | Nervous_system | TRUE |
| SRR22904962 | *Drosophila erecta* | Embryo | TRUE |  | SRR10161423 | *Helicorthomorpha holstii* | Embryo | TRUE |
| SRR22904978 | *Drosophila erecta* | Female_Reproductive_Tissue | TRUE |  | SRR10161414 | *Helicorthomorpha holstii* | Whole_body | TRUE |
| SRR22904979 | *Drosophila erecta* | Female_Reproductive_Tissue | TRUE |  | SRR10161415 | *Helicorthomorpha holstii* | Whole_body | TRUE |
| SRR22905017 | *Drosophila erecta* | Female_Reproductive_Tissue | TRUE |  | SRR10161417 | *Helicorthomorpha holstii* | Whole_body | TRUE |
| SRR22905024 | *Drosophila erecta* | Female_Reproductive_Tissue | TRUE |  | SRR10161422 | *Helicorthomorpha holstii* | Whole_body | TRUE |
| SRR6667443 | *Drosophila erecta* | Male_Reproductive_Tissue | TRUE |  | SRR13625116 | *Helicoverpa armigera* | Whole_body | TRUE |
| SRR24714834 | *Drosophila erecta* | Whole_body | TRUE |  | SRR13625117 | *Helicoverpa armigera* | Whole_body | TRUE |
| SRR24714835 | *Drosophila erecta* | Whole_body | TRUE |  | SRR13625108 | *Helicoverpa armigera* | Whole_body | TRUE |
| SRR24714836 | *Drosophila erecta* | Whole_body | TRUE |  | SRR13625109 | *Helicoverpa armigera* | Whole_body | TRUE |
| SRR24714839 | *Drosophila erecta* | Whole_body | FALSE |  | SRR6219822 | *Scylla paramamosain* | Female_Reproductive_Tissue | TRUE |
| SRR5687198 | *Drosophila melanogaster* | Embryo | TRUE |  | SRR6219825 | *Scylla paramamosain* | Female_Reproductive_Tissue | TRUE |
| SRR5687200 | *Drosophila melanogaster* | Embryo | TRUE |  | SRR6219827 | *Scylla paramamosain* | Female_Reproductive_Tissue | TRUE |
| SRR5687210 | *Drosophila melanogaster* | Embryo | TRUE |  | SRR6219729 | *Scylla paramamosain* | Male_Reproductive_Tissue | FALSE |
| SRR21452132 | *Penaeus vannamei* | Female_Reproductive_Tissue | TRUE |  | SRR6219790 | *Scylla paramamosain* | Male_Reproductive_Tissue | FALSE |
| SRR21452133 | *Penaeus vannamei* | Female_Reproductive_Tissue | TRUE |  | SRR6219809 | *Scylla paramamosain* | Male_Reproductive_Tissue | FALSE |
| SRR21452134 | *Penaeus vannamei* | Female_Reproductive_Tissue | TRUE |  | SRR16323526 | *Scylla paramamosain* | Whole_body | FALSE |
| SRR21452129 | *Penaeus vannamei* | Male_Reproductive_Tissue | TRUE |  | SRR16323527 | *Scylla paramamosain* | Whole_body | FALSE |
| SRR21452130 | *Penaeus vannamei* | Male_Reproductive_Tissue | TRUE |  | SRR16323532 | *Scylla paramamosain* | Whole_body | FALSE |
| SRR21452131 | *Penaeus vannamei* | Male_Reproductive_Tissue | TRUE |  | SRR16323533 | *Scylla paramamosain* | Whole_body | FALSE |
| SRR1552694 | *Penaeus vannamei* | Other_somatic_tissues | FALSE |  | SRR13083967 | *Tribolium castaneum* | Whole_body | TRUE |
| SRR10015080 | *Periplaneta americana* | Whole_body | TRUE |  | SRR13083968 | *Tribolium castaneum* | Whole_body | TRUE |
| SRR10015081 | *Periplaneta americana* | Whole_body | TRUE |  | SRR3479030 | *Tribolium castaneum* | Whole_body | TRUE |
| SRR10015082 | *Periplaneta americana* | Whole_body | TRUE |  |  |  |  |  |
| SRR10015083 | *Periplaneta americana* | Whole_body | TRUE |  |  |  |  |  |

Supplementary Table S3**:** Number of genes belonging to each of the four *Argonaute* sub-families, *AGO1*, *AGO2,* *AGO3*, and *piwi* (including flies *aub*) identified in each species genome annotation.

| Accession | Species | AGO1 | AGO2 | AGO3 | PIWI |
| --- | --- | --- | --- | --- | --- |
| GCF_000142985.2 | *Acyrthosiphon pisum* | 2 | 1 | 3 | 12 |
| GCF_002204515.2 | *Aedes aegypti* | 1 |  | 1 | 7 |
| GCF_035046485.1 | *Aedes albopictus* | 1 |  | 1 | 6 |
| GCF_029169275.1 | *Apis cerana* | 1 | 1 | 1 | 1 |
| GCF_003254395.2 | *Apis mellifera* | 1 | 1 | 1 | 1 |
| GCA_009739505.2 | *Apolygus lucorum* | 1 | 1 | 2 | 1 |
| GCA_003018175.1 | *Blattella germanica* | 1 | 1 | 1 | 2 |
| GCF_030269925.1 | *Bombyx mori* | 1 | 1 | 1 | 1 |
| GCF_000005135.1 | *Drosophila erecta* | 1 | 1 | 1 | 2 |
| GCA_000001215.4 | *Drosophila melanogaster* | 1 | 1 | 1 | 2 |
| GCF_017312745 | *Gryllus bimaculatus* | 1 | 1 | 3 | 4 |
| So et al. (2022) [7] - FigShare | *Helicorthomorpha holstii* | 1 | 1 | 1 | 2 |
| GCF_030705265.1 | *Helicoverpa armigera* | 1 | 1 | 1 | 2 |
| GCA_003789085.1 | *Penaeus vannamei* | 1 | 1 | 1 |  |
| GCA_025594305.2 | *Periplaneta americana* | 1 |  |  | 1 |
| GCA_000002335.3 | *Tribolium castaneum* | 2 | 2 | 1 | 1 |
| GCF_035594125.1 | *Scylla paramamosain* | 1 | 4 | 1 | 2 |

# Supplementary References

1 Andrews S (2010) FastQC: a quality control tool for high throughput sequence data. .

2 Langmead B & Salzberg SL (2012) Fast gapped-read alignment with Bowtie 2. *Nat Methods* **9**, 357–9.

3 Pertea M & Pertea G (2020) GFF Utilities: GffRead and GffCompare. *F1000Res* **9**, 304.

4 Kulkarni A, Ewen-Campen B, Terao K, Matsumoto Y, Li Y, Watanabe T, Kao JA, Parhad SS, Ylla G, Mizunami M & Extavour CG (2023) oskar acts with the transcription factor Creb to regulate long-term memory in crickets. *Proc Natl Acad Sci U S A* **120**, e2218506120.

5 Edgar RC (2004) MUSCLE: multiple sequence alignment with high accuracy and high throughput. *Nucleic Acids Res* **32**, 1792–7.

6 Stamatakis A (2014) RAxML version 8: a tool for phylogenetic analysis and post-analysis of large phylogenies. *Bioinformatics* **30**, 1312–1313.

7 So WL, Nong W, Xie Y, Baril T, Ma H yao, Qu Z, Haimovitz J, Swale T, Gaitan-Espitia JD, Lau KF, Tobe SS, Bendena WG, Kai Z peng, Hayward A & Hui JHL (2022) Myriapod genomes reveal ancestral horizontal gene transfer and hormonal gene loss in millipedes. *Nature Communications 2022 13:1* **13**, 1–12.
